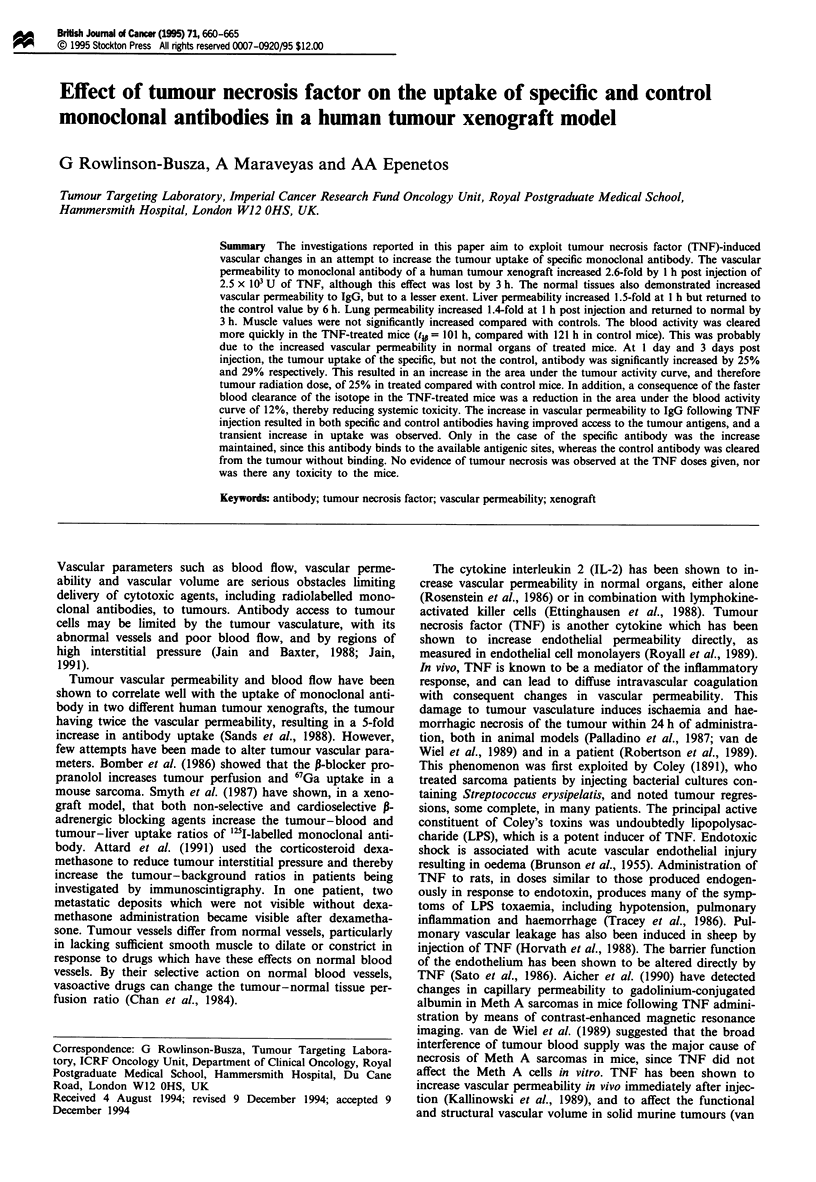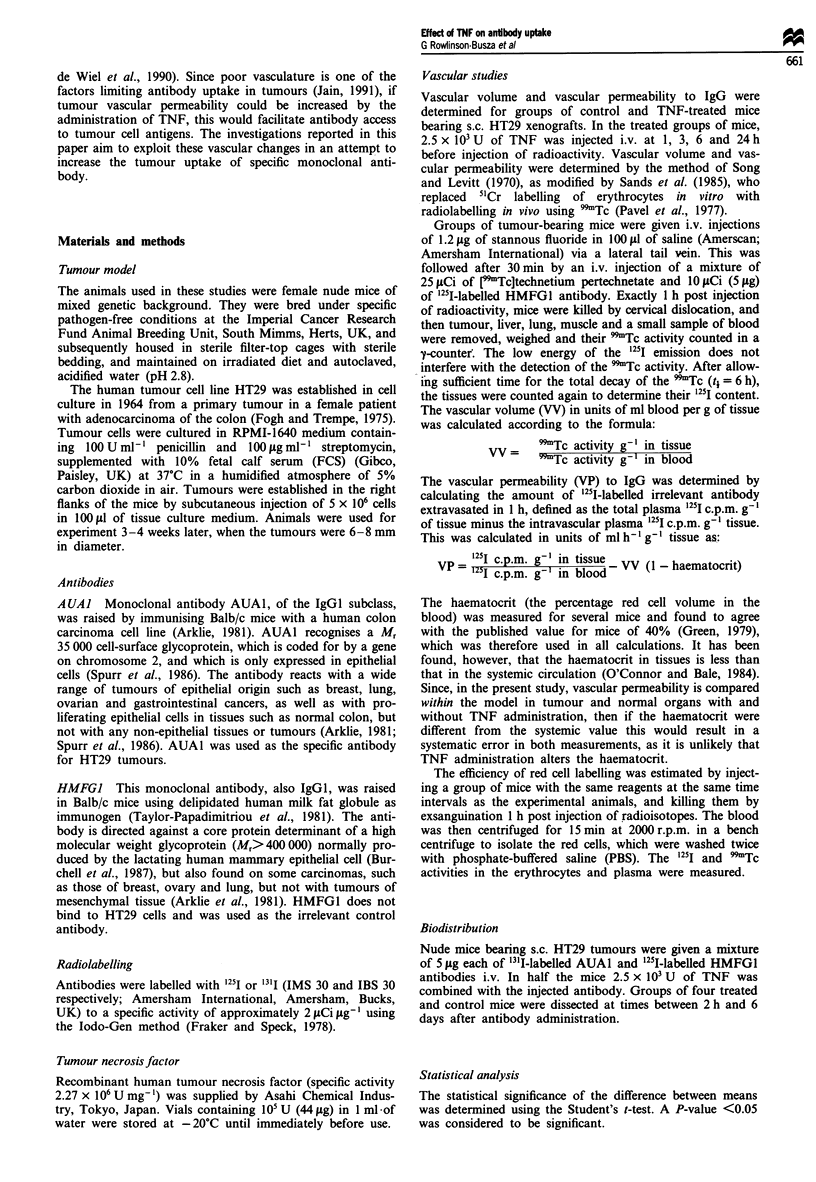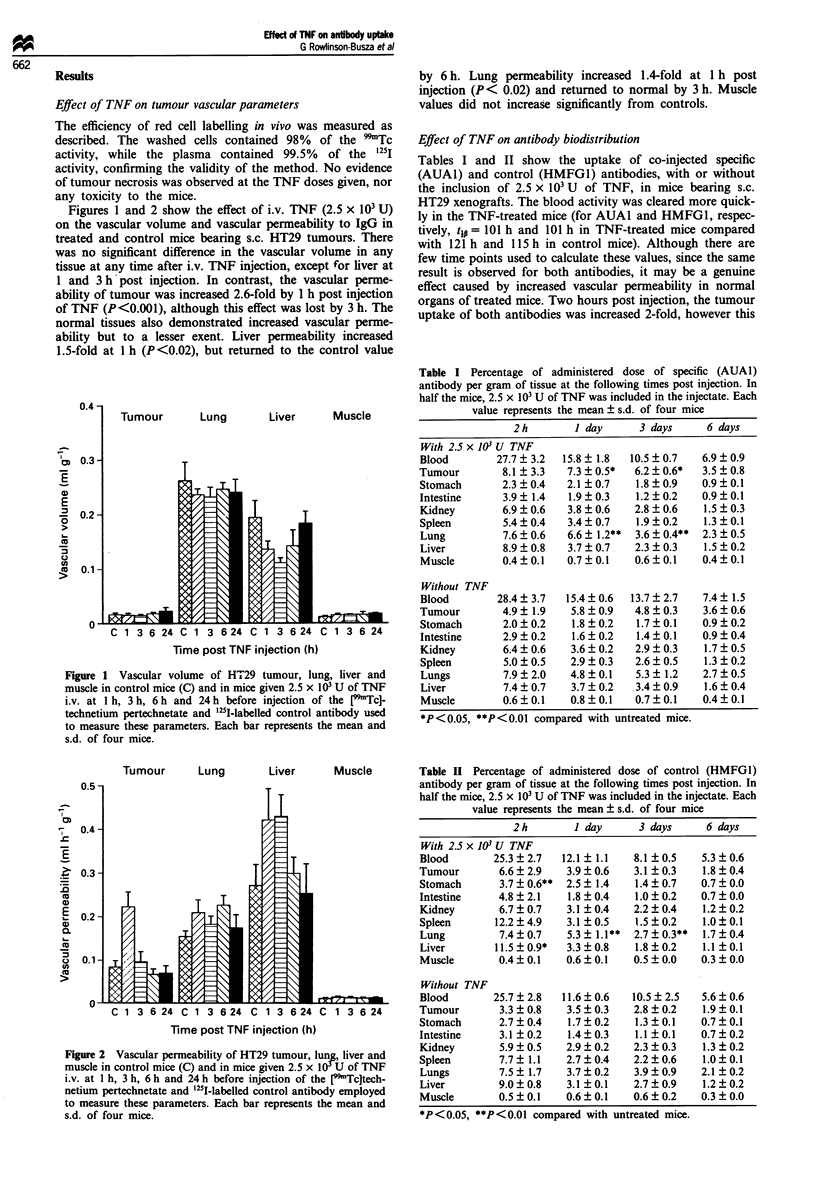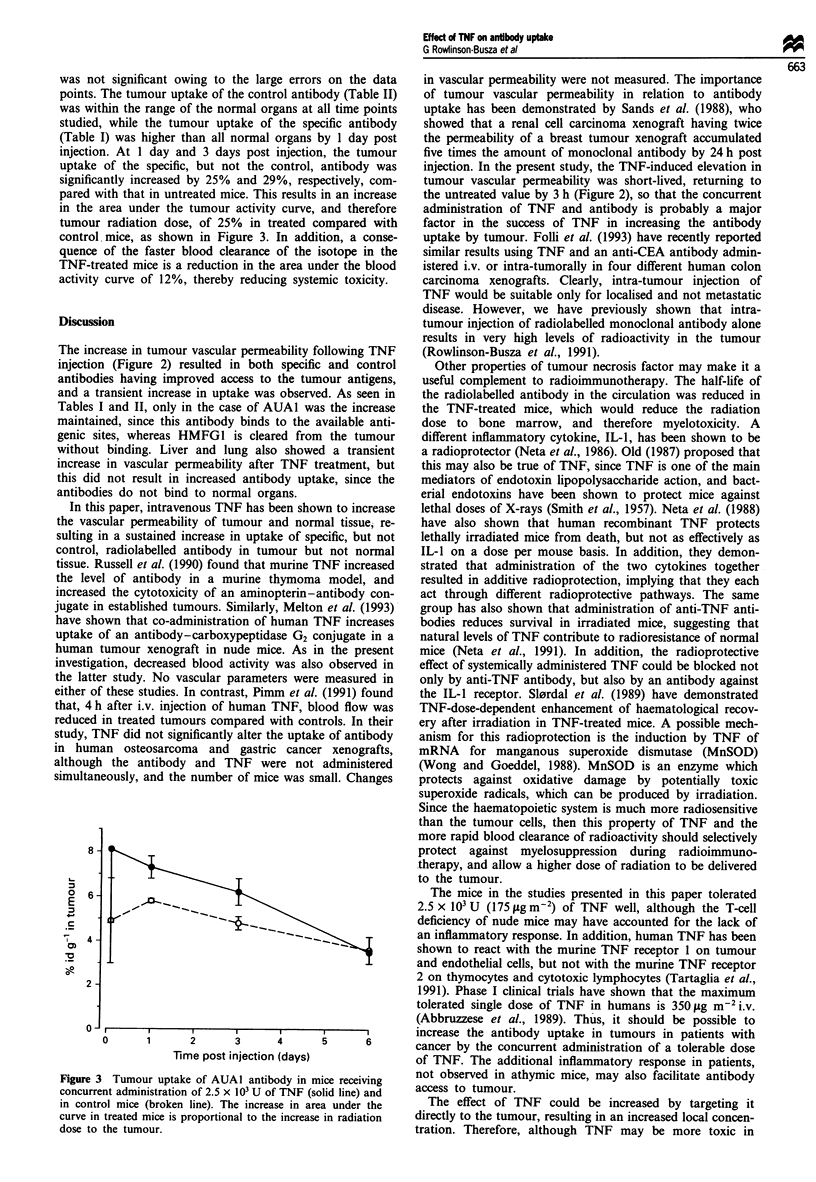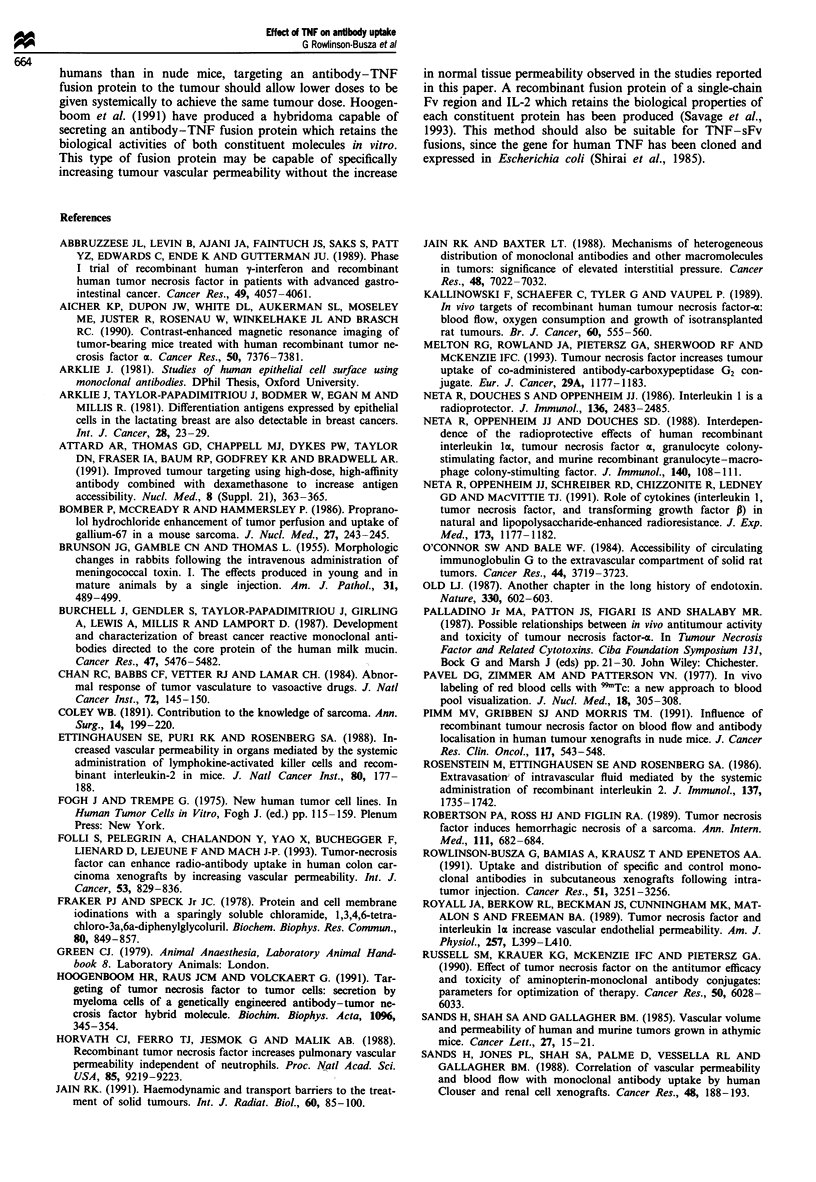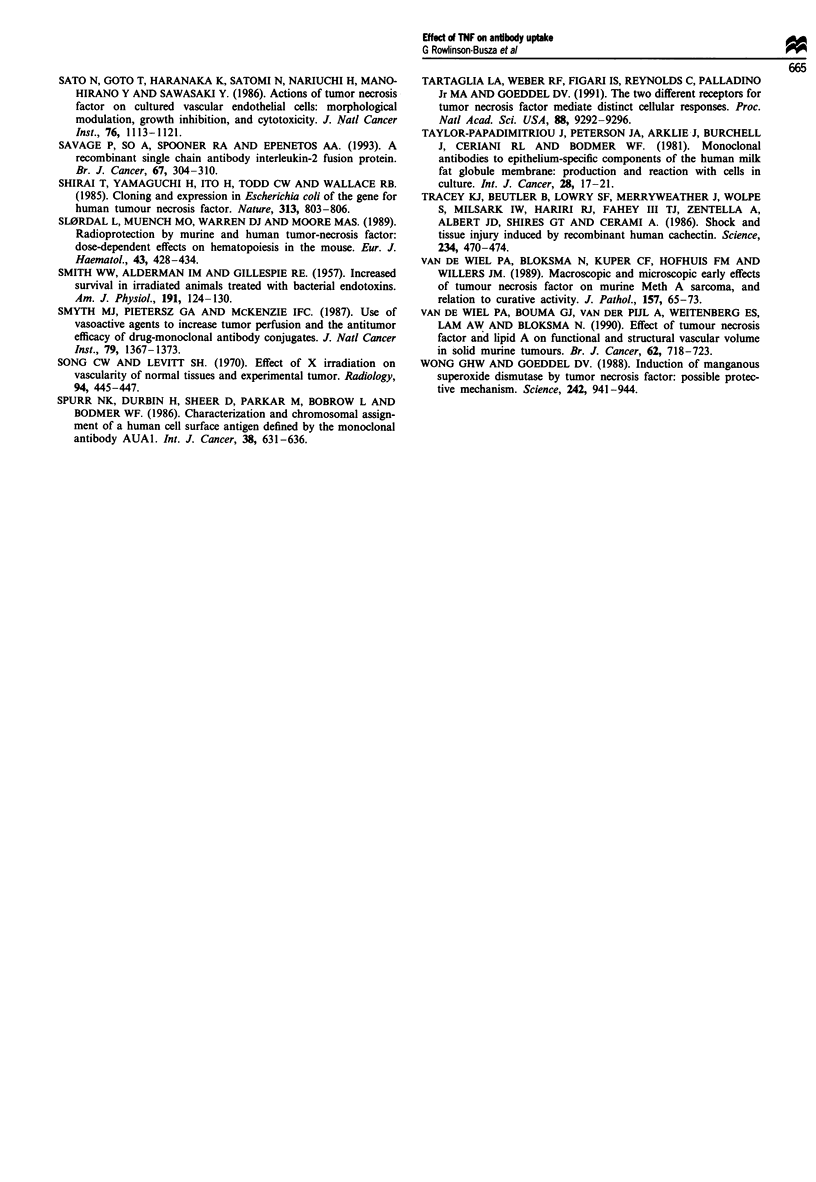# Effect of tumour necrosis factor on the uptake of specific and control monoclonal antibodies in a human tumour xenograft model.

**DOI:** 10.1038/bjc.1995.131

**Published:** 1995-04

**Authors:** G. Rowlinson-Busza, A. Maraveyas, A. A. Epenetos

**Affiliations:** Imperial Cancer Research Fund Oncology Unit, Royal Postgraduate Medical School, Hammersmith Hospital, London, UK.

## Abstract

The investigations reported in this paper aim to exploit tumour necrosis factor (TNF)-induced vascular changes in an attempt to increase the tumour uptake of specific monoclonal antibody. The vascular permeability to monoclonal antibody of a human tumour xenograft increased 2.6-fold by 1 h post injection of 2.5 x 10(3) U of TNF, although this effect was lost by 3 h. The normal tissues also demonstrated increased vascular permeability to IgG, but to a lesser extent. Liver permeability increased 1.5-fold at 1 h but returned to the control value by 6 h. Lung permeability increased 1.4-fold at 1 h post injection and returned to normal by 3 h. Muscle values were not significantly increased compared with controls. The blood activity was cleared more quickly in the TNF-treated mice (t1/2 beta = 101 h, compared with 121 h in control mice). This was probably due to the increased vascular permeability in normal organs of treated mice. At 1 day and 3 days post injection, the tumour uptake of the specific, but not the control, antibody was significantly increased by 25% and 29% respectively. This resulted in an increase in the area under the tumour activity curve, and therefore tumour radiation dose, of 25% in treated compared with control mice. In addition, a consequence of the faster blood clearance of the isotope in the TNF-treated mice was a reduction in the area under the blood activity curve of 12%, thereby reducing systemic toxicity. The increase in vascular permeability to IgG following TNF injection resulted in both specific and control antibodies having improved access to the tumour antigens, and a transient increase in uptake was observed. Only in the case of the specific antibody was the increase maintained, since this antibody binds to the available antigenic sites, whereas the control antibody was cleared from the tumour without binding. No evidence of tumour necrosis was observed at the TNF doses given, nor was there any toxicity to the mice.


British Journal of Cancer (1995) 71, 660-665
© 1995 Stockton Press All rights reserved 0007-0920/95 $12.00

### Effect of tumour necrosis factor on the uptake of specific and control monoclonal antibodies in a human tumour xenograft model

G Rowlinson-Busza, A Maraveyas and AA Epenetos

*Tumour Targeting Laboratory, Imperial Cancer Research Fund Oncology Unit, Royal Postgraduate Medical School, Hammersmith Hospital, London W12 0HS, UK.*

**Summary** The investigations reported in this paper aim to exploit tumour necrosis factor (TNF)-induced vascular changes in an attempt to increase the tumour uptake of specific monoclonal antibody. The vascular permeability to monoclonal antibody of a human tumour xenograft increased 2.6-fold by 1 h post injection of $2.5 \times 10^3$ U of TNF, although this effect was lost by 3 h. The normal tissues also demonstrated increased vascular permeability to IgG, but to a lesser exent. Liver permeability increased 1.5-fold at 1 h but returned to the control value by 6 h. Lung permeability increased 1.4-fold at 1 h post injection and returned to normal by 3 h. Muscle values were not significantly increased compared with controls. The blood activity was cleared more quickly in the TNF-treated mice ($t_{1/2\beta}$ = 101 h, compared with 121 h in control mice). This was probably due to the increased vascular permeability in normal organs of treated mice. At 1 day and 3 days post injection, the tumour uptake of the specific, but not the control, antibody was significantly increased by 25% and 29% respectively. This resulted in an increase in the area under the tumour activity curve, and therefore tumour radiation dose, of 25% in treated compared with control mice. In addition, a consequence of the faster blood clearance of the isotope in the TNF-treated mice was a reduction in the area under the blood activity curve of 12%, thereby reducing systemic toxicity. The increase in vascular permeability to IgG following TNF injection resulted in both specific and control antibodies having improved access to the tumour antigens, and a transient increase in uptake was observed. Only in the case of the specific antibody was the increase maintained, since this antibody binds to the available antigenic sites, whereas the control antibody was cleared from the tumour without binding. No evidence of tumour necrosis was observed at the TNF doses given, nor was there any toxicity to the mice.

**Keywords:** antibody; tumour necrosis factor; vascular permeability; xenograft

Vascular parameters such as blood flow, vascular permeability and vascular volume are serious obstacles limiting delivery of cytotoxic agents, including radiolabelled monoclonal antibodies, to tumours. Antibody access to tumour cells may be limited by the tumour vasculature, with its abnormal vessels and poor blood flow, and by regions of high interstitial pressure (Jain and Baxter, 1988; Jain, 1991).

Tumour vascular permeability and blood flow have been shown to correlate well with the uptake of monoclonal antibody in two different human tumour xenografts, the tumour having twice the vascular permeability, resulting in a 5-fold increase in antibody uptake (Sands *et al.*, 1988). However, few attempts have been made to alter tumour vascular parameters. Bomber *et al.* (1986) showed that the β-blocker propranolol increases tumour perfusion and $^{67}$Ga uptake in a mouse sarcoma. Smyth *et al.* (1987) have shown, in a xenograft model, that both non-selective and cardioselective β-adrenergic blocking agents increase the tumour-blood and tumour-liver uptake ratios of $^{125}$I-labelled monoclonal antibody. Attard *et al.* (1991) used the corticosteroid dexamethasone to reduce tumour interstitial pressure and thereby increase the tumour-background ratios in patients being investigated by immunoscintigraphy. In one patient, two metastatic deposits which were not visible without dexamethasone administration became visible after dexamethasone. Tumour vessels differ from normal vessels, particularly in lacking sufficient smooth muscle to dilate or constrict in response to drugs which have these effects on normal blood vessels. By their selective action on normal blood vessels, vasoactive drugs can change the tumour-normal tissue perfusion ratio (Chan *et al.*, 1984).

The cytokine interleukin 2 (IL-2) has been shown to increase vascular permeability in normal organs, either alone (Rosenstein *et al.*, 1986) or in combination with lymphokine-activated killer cells (Ettinghausen *et al.*, 1988). Tumour necrosis factor (TNF) is another cytokine which has been shown to increase endothelial permeability directly, as measured in endothelial cell monolayers (Royall *et al.*, 1989). *In vivo*, TNF is known to be a mediator of the inflammatory response, and can lead to diffuse intravascular coagulation with consequent changes in vascular permeability. This damage to tumour vasculature induces ischaemia and haemorrhagic necrosis of the tumour within 24 h of administration, both in animal models (Palladino *et al.*, 1987; van de Wiel *et al.*, 1989) and in a patient (Robertson *et al.*, 1989). This phenomenon was first exploited by Coley (1891), who treated sarcoma patients by injecting bacterial cultures containing *Streptococcus erysipelatis*, and noted tumour regressions, some complete, in many patients. The principal active constituent of Coley's toxins was undoubtedly lipopolysaccharide (LPS), which is a potent inducer of TNF. Endotoxic shock is associated with acute vascular endothelial injury resulting in oedema (Brunson *et al.*, 1955). Administration of TNF to rats, in doses similar to those produced endogenously in response to endotoxin, produces many of the symptoms of LPS toxaemia, including hypotension, pulmonary inflammation and haemorrhage (Tracey *et al.*, 1986). Pulmonary vascular leakage has also been induced in sheep by injection of TNF (Horvath *et al.*, 1988). The barrier function of the endothelium has been shown to be altered directly by TNF (Sato *et al.*, 1986). Aicher *et al.* (1990) have detected changes in capillary permeability to gadolinium-conjugated albumin in Meth A sarcomas in mice following TNF administration by means of contrast-enhanced magnetic resonance imaging. van de Wiel *et al.* (1989) suggested that the broad interference of tumour blood supply was the major cause of necrosis of Meth A sarcomas in mice, since TNF did not affect the Meth A cells *in vitro*. TNF has been shown to increase vascular permeability *in vivo* immediately after injection (Kallinowski *et al.*, 1989), and to affect the functional and structural vascular volume in solid murine tumours (van

Correspondence: G Rowlinson-Busza, Tumour Targeting Laboratory, ICRF Oncology Unit, Department of Clinical Oncology, Royal Postgraduate Medical School, Hammersmith Hospital, Du Cane Road, London W12 0HS, UK
Received 4 August 1994; revised 9 December 1994; accepted 9 December 1994

de Wiel et al., 1990). Since poor vasculature is one of the factors limiting antibody uptake in tumours (Jain, 1991), if tumour vascular permeability could be increased by the administration of TNF, this would facilitate antibody access to tumour cell antigens. The investigations reported in this paper aim to exploit these vascular changes in an attempt to increase the tumour uptake of specific monoclonal antibody.

### Materials and methods

### *Tumour model*

The animals used in these studies were female nude mice of mixed genetic background. They were bred under specific pathogen-free conditions at the Imperial Cancer Research Fund Animal Breeding Unit, South Mimms, Herts, UK, and subsequently housed in sterile filter-top cages with sterile bedding, and maintained on irradiated diet and autoclaved, acidified water (pH 2.8).

The human tumour cell line HT29 was established in cell culture in 1964 from a primary tumour in a female patient with adenocarcinoma of the colon (Fogh and Trempe, 1975). Tumour cells were cultured in RPMI-1640 medium containing 100 U $ml^{-1}$ penicillin and 100 $\mu g\ ml^{-1}$ streptomycin, supplemented with 10% fetal calf serum (FCS) (Gibco, Paisley, UK) at 37°C in a humidified atmosphere of 5% carbon dioxide in air. Tumours were established in the right flanks of the mice by subcutaneous injection of $5 \times 10^6$ cells in 100 $\mu l$ of tissue culture medium. Animals were used for experiment 3–4 weeks later, when the tumours were 6–8 mm in diameter.

### *Antibodies*

*AUA1* Monoclonal antibody AUA1, of the IgG1 subclass, was raised by immunising Balb/c mice with a human colon carcinoma cell line (Arklie, 1981). AUA1 recognises a $M_r$ 35 000 cell-surface glycoprotein, which is coded for by a gene on chromosome 2, and which is only expressed in epithelial cells (Spurr et al., 1986). The antibody reacts with a wide range of tumours of epithelial origin such as breast, lung, ovarian and gastrointestinal cancers, as well as with proliferating epithelial cells in tissues such as normal colon, but not with any non-epithelial tissues or tumours (Arklie, 1981; Spurr et al., 1986). AUA1 was used as the specific antibody for HT29 tumours.

*HMFG1* This monoclonal antibody, also IgG1, was raised in Balb/c mice using delipidated human milk fat globule as immunogen (Taylor-Papadimitriou et al., 1981). The antibody is directed against a core protein determinant of a high molecular weight glycoprotein ($M_r$> 400 000) normally produced by the lactating human mammary epithelial cell (Burchell et al., 1987), but also found on some carcinomas, such as those of breast, ovary and lung, but not with tumours of mesenchymal tissue (Arklie et al., 1981). HMFG1 does not bind to HT29 cells and was used as the irrelevant control antibody.

### *Radiolabelling*

Antibodies were labelled with $^{125}I$ or $^{131}I$ (IMS 30 and IBS 30 respectively; Amersham International, Amersham, Bucks, UK) to a specific activity of approximately 2 $\mu Ci\ \mu g^{-1}$ using the Iodo-Gen method (Fraker and Speck, 1978).

### *Tumour necrosis factor*

Recombinant human tumour necrosis factor (specific activity $2.27 \times 10^6$ U $mg^{-1}$) was supplied by Asahi Chemical Industry, Tokyo, Japan. Vials containing $10^5$ U (44 $\mu g$) in 1 ml of water were stored at −20°C until immediately before use.

### *Vascular studies*

Vascular volume and vascular permeability to IgG were determined for groups of control and TNF-treated mice bearing s.c. HT29 xenografts. In the treated groups of mice, $2.5 \times 10^3$ U of TNF was injected i.v. at 1, 3, 6 and 24 h before injection of radioactivity. Vascular volume and vascular permeability were determined by the method of Song and Levitt (1970), as modified by Sands et al. (1985), who replaced $^{51}Cr$ labelling of erythrocytes *in vitro* with radiolabelling *in vivo* using $^{99m}Tc$ (Pavel et al., 1977).

Groups of tumour-bearing mice were given i.v. injections of 1.2 $\mu g$ of stannous fluoride in 100 $\mu l$ of saline (Amerscan; Amersham International) via a lateral tail vein. This was followed after 30 min by an i.v. injection of a mixture of 25 $\mu Ci$ of [$^{99m}Tc$]technetium pertechnetate and 10 $\mu Ci$ (5 $\mu g$) of $^{125}I$-labelled HMFG1 antibody. Exactly 1 h post injection of radioactivity, mice were killed by cervical dislocation, and then tumour, liver, lung, muscle and a small sample of blood were removed, weighed and their $^{99m}Tc$ activity counted in a γ-counter. The low energy of the $^{125}I$ emission does not interfere with the detection of the $^{99m}Tc$ activity. After allowing sufficient time for the total decay of the $^{99m}Tc$ ($t_{\frac{1}{2}} = 6$ h), the tissues were counted again to determine their $^{125}I$ content. The vascular volume (VV) in units of ml blood per g of tissue was calculated according to the formula:

$$VV = \frac{^{99m}Tc\ \text{activity}\ g^{-1}\ \text{in tissue}}{^{99m}Tc\ \text{activity}\ g^{-1}\ \text{in blood}}$$

The vascular permeability (VP) to IgG was determined by calculating the amount of $^{125}I$-labelled irrelevant antibody extravasated in 1 h, defined as the total plasma $^{125}I$ c.p.m. $g^{-1}$ of tissue minus the intravascular plasma $^{125}I$ c.p.m. $g^{-1}$ tissue. This was calculated in units of ml $h^{-1}$ $g^{-1}$ tissue as:

$$VP = \frac{^{125}I\ \text{c.p.m.}\ g^{-1}\ \text{in tissue}}{^{125}I\ \text{c.p.m.}\ g^{-1}\ \text{in blood}} - VV\ (1 - \text{haematocrit})$$

The haematocrit (the percentage red cell volume in the blood) was measured for several mice and found to agree with the published value for mice of 40% (Green, 1979), which was therefore used in all calculations. It has been found, however, that the haematocrit in tissues is less than that in the systemic circulation (O'Connor and Bale, 1984). Since, in the present study, vascular permeability is compared *within* the model in tumour and normal organs with and without TNF administration, then if the haematocrit were different from the systemic value this would result in a systematic error in both measurements, as it is unlikely that TNF administration alters the haematocrit.

The efficiency of red cell labelling was estimated by injecting a group of mice with the same reagents at the same time intervals as the experimental animals, and killing them by exsanguination 1 h post injection of radioisotopes. The blood was then centrifuged for 15 min at 2000 r.p.m. in a bench centrifuge to isolate the red cells, which were washed twice with phosphate-buffered saline (PBS). The $^{125}I$ and $^{99m}Tc$ activities in the erythrocytes and plasma were measured.

### *Biodistribution*

Nude mice bearing s.c. HT29 tumours were given a mixture of 5 $\mu g$ each of $^{131}I$-labelled AUA1 and $^{125}I$-labelled HMFG1 antibodies i.v. In half the mice $2.5 \times 10^3$ U of TNF was combined with the injected antibody. Groups of four treated and control mice were dissected at times between 2 h and 6 days after antibody administration.

### *Statistical analysis*

The statistical significance of the difference between means was determined using the Student's *t*-test. A *P*-value $<0.05$ was considered to be significant.

### Results

### *Effect of TNF on tumour vascular parameters*

The efficiency of red cell labelling *in vivo* was measured as described. The washed cells contained 98% of the $^{99m}$Tc activity, while the plasma contained 99.5% of the $^{125}$I activity, confirming the validity of the method. No evidence of tumour necrosis was observed at the TNF doses given, nor any toxicity to the mice.

Figures 1 and 2 show the effect of i.v. TNF ($2.5 \times 10^3$ U) on the vascular volume and vascular permeability to IgG in treated and control mice bearing s.c. HT29 tumours. There was no significant difference in the vascular volume in any tissue at any time after i.v. TNF injection, except for liver at 1 and 3 h post injection. In contrast, the vascular permeability of tumour was increased 2.6-fold by 1 h post injection of TNF ($P<0.001$), although this effect was lost by 3 h. The normal tissues also demonstrated increased vascular permeability but to a lesser exent. Liver permeability increased 1.5-fold at 1 h ($P<0.02$), but returned to the control value by 6 h. Lung permeability increased 1.4-fold at 1 h post injection ($P< 0.02$) and returned to normal by 3 h. Muscle values did not increase significantly from controls.

**Figure 1** Vascular volume of HT29 tumour, lung, liver and muscle in control mice (C) and in mice given $2.5 \times 10^3$ U of TNF i.v. at 1 h, 3 h, 6 h and 24 h before injection of the [$^{99m}$Tc]-technetium pertechnetate and $^{125}$I-labelled control antibody used to measure these parameters. Each bar represents the mean and s.d. of four mice.

**Figure 2** Vascular permeability of HT29 tumour, lung, liver and muscle in control mice (C) and in mice given $2.5 \times 10^3$ U of TNF i.v. at 1 h, 3 h, 6 h and 24 h before injection of the [$^{99m}$Tc]technetium pertechnetate and $^{125}$I-labelled control antibody employed to measure these parameters. Each bar represents the mean and s.d. of four mice.

### *Effect of TNF on antibody biodistribution*

Tables I and II show the uptake of co-injected specific (AUA1) and control (HMFG1) antibodies, with or without the inclusion of $2.5 \times 10^3$ U of TNF, in mice bearing s.c. HT29 xenografts. The blood activity was cleared more quickly in the TNF-treated mice (for AUA1 and HMFG1, respectively, $t_{\frac{1}{2}\beta} = 101$ h and 101 h in TNF-treated mice compared with 121 h and 115 h in control mice). Although there are few time points used to calculate these values, since the same result is observed for both antibodies, it may be a genuine effect caused by increased vascular permeability in normal organs of treated mice. Two hours post injection, the tumour uptake of both antibodies was increased 2-fold, however this

**Table I** Percentage of administered dose of specific (AUA1) antibody per gram of tissue at the following times post injection. In half the mice, $2.5 \times 10^3$ U of TNF was included in the injectate. Each value represents the mean ± s.d. of four mice

| | *2 h* | *1 day* | *3 days* | *6 days* |
|---|---|---|---|---|
| *With 2.5 × 10³ U TNF* | | | | |
| Blood | 27.7 ± 3.2 | 15.8 ± 1.8 | 10.5 ± 0.7 | 6.9 ± 0.9 |
| Tumour | 8.1 ± 3.3 | 7.3 ± 0.5* | 6.2 ± 0.6* | 3.5 ± 0.8 |
| Stomach | 2.3 ± 0.4 | 2.1 ± 0.7 | 1.8 ± 0.9 | 0.9 ± 0.1 |
| Intestine | 3.9 ± 1.4 | 1.9 ± 0.3 | 1.2 ± 0.2 | 0.9 ± 0.1 |
| Kidney | 6.9 ± 0.6 | 3.8 ± 0.6 | 2.8 ± 0.6 | 1.5 ± 0.3 |
| Spleen | 5.4 ± 0.4 | 3.4 ± 0.7 | 1.9 ± 0.2 | 1.3 ± 0.1 |
| Lung | 7.6 ± 0.6 | 6.6 ± 1.2** | 3.6 ± 0.4** | 2.3 ± 0.5 |
| Liver | 8.9 ± 0.8 | 3.7 ± 0.7 | 2.3 ± 0.3 | 1.5 ± 0.2 |
| Muscle | 0.4 ± 0.1 | 0.7 ± 0.1 | 0.6 ± 0.1 | 0.4 ± 0.1 |
| *Without TNF* | | | | |
| Blood | 28.4 ± 3.7 | 15.4 ± 0.6 | 13.7 ± 2.7 | 7.4 ± 1.5 |
| Tumour | 4.9 ± 1.9 | 5.8 ± 0.9 | 4.8 ± 0.3 | 3.6 ± 0.6 |
| Stomach | 2.0 ± 0.2 | 1.8 ± 0.2 | 1.7 ± 0.1 | 0.9 ± 0.2 |
| Intestine | 2.9 ± 0.2 | 1.6 ± 0.2 | 1.4 ± 0.1 | 0.9 ± 0.4 |
| Kidney | 6.4 ± 0.6 | 3.6 ± 0.2 | 2.9 ± 0.3 | 1.7 ± 0.5 |
| Spleen | 5.0 ± 0.5 | 2.9 ± 0.3 | 2.6 ± 0.5 | 1.3 ± 0.2 |
| Lungs | 7.9 ± 2.0 | 4.8 ± 0.1 | 5.3 ± 1.2 | 2.7 ± 0.5 |
| Liver | 7.4 ± 0.7 | 3.7 ± 0.2 | 3.4 ± 0.9 | 1.6 ± 0.4 |
| Muscle | 0.6 ± 0.1 | 0.8 ± 0.1 | 0.7 ± 0.1 | 0.4 ± 0.1 |

*$P<0.05$, **$P<0.01$ compared with untreated mice.

**Table II** Percentage of administered dose of control (HMFG1) antibody per gram of tissue at the following times post injection. In half the mice, $2.5 \times 10^3$ U of TNF was included in the injectate. Each value represents the mean ± s.d. of four mice

| | *2 h* | *1 day* | *3 days* | *6 days* |
|---|---|---|---|---|
| *With 2.5 × 10³ U TNF* | | | | |
| Blood | 25.3 ± 2.7 | 12.1 ± 1.1 | 8.1 ± 0.5 | 5.3 ± 0.6 |
| Tumour | 6.6 ± 2.9 | 3.9 ± 0.6 | 3.1 ± 0.3 | 1.8 ± 0.4 |
| Stomach | 3.7 ± 0.6** | 2.5 ± 1.4 | 1.4 ± 0.7 | 0.7 ± 0.0 |
| Intestine | 4.8 ± 2.1 | 1.8 ± 0.4 | 1.0 ± 0.2 | 0.7 ± 0.0 |
| Kidney | 6.7 ± 0.7 | 3.1 ± 0.4 | 2.2 ± 0.4 | 1.2 ± 0.2 |
| Spleen | 12.2 ± 4.9 | 3.1 ± 0.5 | 1.5 ± 0.2 | 1.0 ± 0.1 |
| Lung | 7.4 ± 0.7 | 5.3 ± 1.1** | 2.7 ± 0.3** | 1.7 ± 0.4 |
| Liver | 11.5 ± 0.9* | 3.3 ± 0.8 | 1.8 ± 0.2 | 1.1 ± 0.1 |
| Muscle | 0.4 ± 0.1 | 0.6 ± 0.1 | 0.5 ± 0.0 | 0.3 ± 0.0 |
| *Without TNF* | | | | |
| Blood | 25.7 ± 2.8 | 11.6 ± 0.6 | 10.5 ± 2.5 | 5.6 ± 0.6 |
| Tumour | 3.3 ± 0.8 | 3.5 ± 0.3 | 2.8 ± 0.2 | 1.9 ± 0.1 |
| Stomach | 2.7 ± 0.4 | 1.7 ± 0.2 | 1.3 ± 0.1 | 0.7 ± 0.1 |
| Intestine | 3.1 ± 0.2 | 1.4 ± 0.3 | 1.1 ± 0.1 | 0.7 ± 0.2 |
| Kidney | 5.9 ± 0.5 | 2.9 ± 0.2 | 2.3 ± 0.3 | 1.3 ± 0.2 |
| Spleen | 7.7 ± 1.1 | 2.7 ± 0.4 | 2.2 ± 0.6 | 1.0 ± 0.1 |
| Lungs | 7.5 ± 1.7 | 3.7 ± 0.2 | 3.9 ± 0.9 | 2.1 ± 0.2 |
| Liver | 9.0 ± 0.8 | 3.1 ± 0.1 | 2.7 ± 0.9 | 1.2 ± 0.2 |
| Muscle | 0.5 ± 0.1 | 0.6 ± 0.1 | 0.6 ± 0.2 | 0.3 ± 0.0 |

*$P<0.05$, **$P<0.01$ compared with untreated mice.

was not significant owing to the large errors on the data points. The tumour uptake of the control antibody (Table II) was within the range of the normal organs at all time points studied, while the tumour uptake of the specific antibody (Table I) was higher than all normal organs by 1 day post injection. At 1 day and 3 days post injection, the tumour uptake of the specific, but not the control, antibody was significantly increased by 25% and 29%, respectively, compared with that in untreated mice. This results in an increase in the area under the tumour activity curve, and therefore tumour radiation dose, of 25% in treated compared with control mice, as shown in Figure 3. In addition, a consequence of the faster blood clearance of the isotope in the TNF-treated mice is a reduction in the area under the blood activity curve of 12%, thereby reducing systemic toxicity.

### Discussion

The increase in tumour vascular permeability following TNF injection (Figure 2) resulted in both specific and control antibodies having improved access to the tumour antigens, and a transient increase in uptake was observed. As seen in Tables I and II, only in the case of AUA1 was the increase maintained, since this antibody binds to the available antigenic sites, whereas HMFG1 is cleared from the tumour without binding. Liver and lung also showed a transient increase in vascular permeability after TNF treatment, but this did not result in increased antibody uptake, since the antibodies do not bind to normal organs.

In this paper, intravenous TNF has been shown to increase the vascular permeability of tumour and normal tissue, resulting in a sustained increase in uptake of specific, but not control, radiolabelled antibody in tumour but not normal tissue. Russell *et al.* (1990) found that murine TNF increased the level of antibody in a murine thymoma model, and increased the cytotoxicity of an aminopterin–antibody conjugate in established tumours. Similarly, Melton *et al.* (1993) have shown that co-administration of human TNF increases uptake of an antibody–carboxypeptidase $G_2$ conjugate in a human tumour xenograft in nude mice. As in the present investigation, decreased blood activity was also observed in the latter study. No vascular parameters were measured in either of these studies. In contrast, Pimm *et al.* (1991) found that, 4 h after i.v. injection of human TNF, blood flow was reduced in treated tumours compared with controls. In their study, TNF did not significantly alter the uptake of antibody in human osteosarcoma and gastric cancer xenografts, although the antibody and TNF were not administered simultaneously, and the number of mice was small. Changes

**Figure 3** Tumour uptake of AUA1 antibody in mice receiving concurrent administration of $2.5 \times 10^3$ U of TNF (solid line) and in control mice (broken line). The increase in area under the curve in treated mice is proportional to the increase in radiation dose to the tumour.

in vascular permeability were not measured. The importance of tumour vascular permeability in relation to antibody uptake has been demonstrated by Sands *et al.* (1988), who showed that a renal cell carcinoma xenograft having twice the permeability of a breast tumour xenograft accumulated five times the amount of monoclonal antibody by 24 h post injection. In the present study, the TNF-induced elevation in tumour vascular permeability was short-lived, returning to the untreated value by 3 h (Figure 2), so that the concurrent administration of TNF and antibody is probably a major factor in the success of TNF in increasing the antibody uptake by tumour. Folli *et al.* (1993) have recently reported similar results using TNF and an anti-CEA antibody administered i.v. or intra-tumorally in four different human colon carcinoma xenografts. Clearly, intra-tumour injection of TNF would be suitable only for localised and not metastatic disease. However, we have previously shown that intra-tumour injection of radiolabelled monoclonal antibody alone results in very high levels of radioactivity in the tumour (Rowlinson-Busza *et al.*, 1991).

Other properties of tumour necrosis factor may make it a useful complement to radioimmunotherapy. The half-life of the radiolabelled antibody in the circulation was reduced in the TNF-treated mice, which would reduce the radiation dose to bone marrow, and therefore myelotoxicity. A different inflammatory cytokine, IL-1, has been shown to be a radioprotector (Neta *et al.*, 1986). Old (1987) proposed that this may also be true of TNF, since TNF is one of the main mediators of endotoxin lipopolysaccharide action, and bacterial endotoxins have been shown to protect mice against lethal doses of X-rays (Smith *et al.*, 1957). Neta *et al.* (1988) have also shown that human recombinant TNF protects lethally irradiated mice from death, but not as effectively as IL-1 on a dose per mouse basis. In addition, they demonstrated that administration of the two cytokines together resulted in additive radioprotection, implying that they each act through different radioprotective pathways. The same group has also shown that administration of anti-TNF antibodies reduces survival in irradiated mice, suggesting that natural levels of TNF contribute to radioresistance of normal mice (Neta *et al.*, 1991). In addition, the radioprotective effect of systemically administered TNF could be blocked not only by anti-TNF antibody, but also by an antibody against the IL-1 receptor. Slørdal *et al.* (1989) have demonstrated TNF-dose-dependent enhancement of haematological recovery after irradiation in TNF-treated mice. A possible mechanism for this radioprotection is the induction by TNF of mRNA for manganous superoxide dismutase (MnSOD) (Wong and Goeddel, 1988). MnSOD is an enzyme which protects against oxidative damage by potentially toxic superoxide radicals, which can be produced by irradiation. Since the haematopoietic system is much more radiosensitive than the tumour cells, then this property of TNF and the more rapid blood clearance of radioactivity should selectively protect against myelosuppression during radioimmunotherapy, and allow a higher dose of radiation to be delivered to the tumour.

The mice in the studies presented in this paper tolerated $2.5 \times 10^3$ U ($175\,\mu g\,m^{-2}$) of TNF well, although the T-cell deficiency of nude mice may have accounted for the lack of an inflammatory response. In addition, human TNF has been shown to react with the murine TNF receptor 1 on tumour and endothelial cells, but not with the murine TNF receptor 2 on thymocytes and cytotoxic lymphocytes (Tartaglia *et al.*, 1991). Phase I clinical trials have shown that the maximum tolerated single dose of TNF in humans is $350\,\mu g\ m^{-2}$ i.v. (Abbruzzese *et al.*, 1989). Thus, it should be possible to increase the antibody uptake in tumours in patients with cancer by the concurrent administration of a tolerable dose of TNF. The additional inflammatory response in patients, not observed in athymic mice, may also facilitate antibody access to tumour.

The effect of TNF could be increased by targeting it directly to the tumour, resulting in an increased local concentration. Therefore, although TNF may be more toxic in humans than in nude mice, targeting an antibody–TNF fusion protein to the tumour should allow lower doses to be given systemically to achieve the same tumour dose. Hoogenboom *et al.* (1991) have produced a hybridoma capable of secreting an antibody–TNF fusion protein which retains the biological activities of both constituent molecules *in vitro*. This type of fusion protein may be capable of specifically increasing tumour vascular permeability without the increase in normal tissue permeability observed in the studies reported in this paper. A recombinant fusion protein of a single-chain Fv region and IL-2 which retains the biological properties of each constituent protein has been produced (Savage *et al.*, 1993). This method should also be suitable for TNF–sFv fusions, since the gene for human TNF has been cloned and expressed in *Escherichia coli* (Shirai *et al.*, 1985).

### References

ABBRUZZESE JL, LEVIN B, AJANI JA, FAINTUCH JS, SAKS S, PATT YZ, EDWARDS C, ENDE K AND GUTTERMAN JU. (1989). Phase I trial of recombinant human γ-interferon and recombinant human tumor necrosis factor in patients with advanced gastrointestinal cancer. *Cancer Res.*, **49**, 4057–4061.

AICHER KP, DUPON JW, WHITE DL, AUKERMAN SL, MOSELEY ME, JUSTER R, ROSENAU W, WINKELHAKE JL AND BRASCH RC. (1990). Contrast-enhanced magnetic resonance imaging of tumor-bearing mice treated with human recombinant tumor necrosis factor α. *Cancer Res.*, **50**, 7376–7381.

ARKLIE J. (1981). *Studies of human epithelial cell surface using monoclonal antibodies.* DPhil Thesis, Oxford University.

ARKLIE J, TAYLOR-PAPADIMITRIOU J, BODMER W, EGAN M AND MILLIS R. (1981). Differentiation antigens expressed by epithelial cells in the lactating breast are also detectable in breast cancers. *Int. J. Cancer*, **28**, 23–29.

ATTARD AR, THOMAS GD, CHAPPELL MJ, DYKES PW, TAYLOR DN, FRASER IA, BAUM RP, GODFREY KR AND BRADWELL AR. (1991). Improved tumour targeting using high-dose, high-affinity antibody combined with dexamethasone to increase antigen accessibility. *Nucl. Med.*, **8** (Suppl. 21), 363–365.

BOMBER P, MCCREADY R AND HAMMERSLEY P. (1986). Propranolol hydrochloride enhancement of tumor perfusion and uptake of gallium-67 in a mouse sarcoma. *J. Nucl. Med.*, **27**, 243–245.

BRUNSON JG, GAMBLE CN AND THOMAS L. (1955). Morphologic changes in rabbits following the intravenous administration of meningococcal toxin. I. The effects produced in young and in mature animals by a single injection. *Am. J. Pathol.*, **31**, 489–499.

BURCHELL J, GENDLER S, TAYLOR-PAPADIMITRIOU J, GIRLING A, LEWIS A, MILLIS R AND LAMPORT D. (1987). Development and characterization of breast cancer reactive monoclonal antibodies directed to the core protein of the human milk mucin. *Cancer Res.*, **47**, 5476–5482.

CHAN RC, BABBS CF, VETTER RJ AND LAMAR CH. (1984). Abnormal response of tumor vasculature to vasoactive drugs. *J. Natl Cancer Inst.*, **72**, 145–150.

COLEY WB. (1891). Contribution to the knowledge of sarcoma. *Ann. Surg.*, **14**, 199–220.

ETTINGHAUSEN SE, PURI RK AND ROSENBERG SA. (1988). Increased vascular permeability in organs mediated by the systemic administration of lymphokine-activated killer cells and recombinant interleukin-2 in mice. *J. Natl Cancer Inst.*, **80**, 177–188.

FOGH J AND TREMPE G. (1975). New human tumor cell lines. In *Human Tumor Cells in Vitro*, Fogh J. (ed.) pp. 115–159. Plenum Press: New York.

FOLLI S, PELEGRIN A, CHALANDON Y, YAO X, BUCHEGGER F, LIENARD D, LEJEUNE F AND MACH J-P. (1993). Tumor-necrosis factor can enhance radio-antibody uptake in human colon carcinoma xenografts by increasing vascular permeability. *Int. J. Cancer*, **53**, 829–836.

FRAKER PJ AND SPECK Jr JC. (1978). Protein and cell membrane iodinations with a sparingly soluble chloramide, 1,3,4,6-tetrachloro-3a,6a-diphenylglycoluril. *Biochem. Biophys. Res. Commun.*, **80**, 849–857.

GREEN CJ. (1979). *Animal Anaesthesia, Laboratory Animal Handbook 8.* Laboratory Animals: London.

HOOGENBOOM HR, RAUS JCM AND VOLCKAERT G. (1991). Targeting of tumor necrosis factor to tumor cells: secretion by myeloma cells of a genetically engineered antibody–tumor necrosis factor hybrid molecule. *Biochim. Biophys. Acta*, **1096**, 345–354.

HORVATH CJ, FERRO TJ, JESMOK G AND MALIK AB. (1988). Recombinant tumor necrosis factor increases pulmonary vascular permeability independent of neutrophils. *Proc. Natl Acad. Sci. USA*, **85**, 9219–9223.

JAIN RK. (1991). Haemodynamic and transport barriers to the treatment of solid tumours. *Int. J. Radiat. Biol.*, **60**, 85–100.

JAIN RK AND BAXTER LT. (1988). Mechanisms of heterogeneous distribution of monoclonal antibodies and other macromolecules in tumors: significance of elevated interstitial pressure. *Cancer Res.*, **48**, 7022–7032.

KALLINOWSKI F, SCHAEFER C, TYLER G AND VAUPEL P. (1989). *In vivo* targets of recombinant human tumour necrosis factor-α: blood flow, oxygen consumption and growth of isotransplanted rat tumours. *Br. J. Cancer*, **60**, 555–560.

MELTON RG, ROWLAND JA, PIETERSZ GA, SHERWOOD RF AND MCKENZIE IFC. (1993). Tumour necrosis factor increases tumour uptake of co-administered antibody-carboxypeptidase $G_2$ conjugate. *Eur. J. Cancer*, **29A**, 1177–1183.

NETA R, DOUCHES S AND OPPENHEIM JJ. (1986). Interleukin 1 is a radioprotector. *J. Immunol.*, **136**, 2483–2485.

NETA R, OPPENHEIM JJ AND DOUCHES SD. (1988). Interdependence of the radioprotective effects of human recombinant interleukin 1α, tumour necrosis factor α, granulocyte colony-stimulating factor, and murine recombinant granulocyte–macrophage colony-stimulting factor. *J. Immunol.*, **140**, 108–111.

NETA R, OPPENHEIM JJ, SCHREIBER RD, CHIZZONITE R, LEDNEY GD AND MACVITTIE TJ. (1991). Role of cytokines (interleukin 1, tumor necrosis factor, and transforming growth factor β) in natural and lipopolysaccharide-enhanced radioresistance. *J. Exp. Med.*, **173**, 1177–1182.

O'CONNOR SW AND BALE WF. (1984). Accessibility of circulating immunoglobulin G to the extravascular compartment of solid rat tumors. *Cancer Res.*, **44**, 3719–3723.

OLD LJ. (1987). Another chapter in the long history of endotoxin. *Nature*, **330**, 602–603.

PALLADINO Jr MA, PATTON JS, FIGARI IS AND SHALABY MR. (1987). Possible relationships between *in vivo* antitumour activity and toxicity of tumour necrosis factor-α. In *Tumour Necrosis Factor and Related Cytotoxins. Ciba Foundation Symposium 131*, Bock G and Marsh J (eds) pp. 21–30. John Wiley: Chichester.

PAVEL DG, ZIMMER AM AND PATTERSON VN. (1977). In vivo labeling of red blood cells with $^{99m}$Tc: a new approach to blood pool visualization. *J. Nucl. Med.*, **18**, 305–308.

PIMM MV, GRIBBEN SJ AND MORRIS TM. (1991). Influence of recombinant tumour necrosis factor on blood flow and antibody localisation in human tumour xenografts in nude mice. *J. Cancer Res. Clin. Oncol.*, **117**, 543–548.

ROSENSTEIN M, ETTINGHAUSEN SE AND ROSENBERG SA. (1986). Extravasation of intravascular fluid mediated by the systemic administration of recombinant interleukin 2. *J. Immunol.*, **137**, 1735–1742.

ROBERTSON PA, ROSS HJ AND FIGLIN RA. (1989). Tumor necrosis factor induces hemorrhagic necrosis of a sarcoma. *Ann. Intern. Med.*, **111**, 682–684.

ROWLINSON-BUSZA G, BAMIAS A, KRAUSZ T AND EPENETOS AA. (1991). Uptake and distribution of specific and control monoclonal antibodies in subcutaneous xenografts following intratumor injection. *Cancer Res.*, **51**, 3251–3256.

ROYALL JA, BERKOW RL, BECKMAN JS, CUNNINGHAM MK, MATALON S AND FREEMAN BA. (1989). Tumor necrosis factor and interleukin 1α increase vascular endothelial permeability. *Am. J. Physiol.*, **257**, L399–L410.

RUSSELL SM, KRAUER KG, MCKENZIE IFC AND PIETERSZ GA. (1990). Effect of tumor necrosis factor on the antitumor efficacy and toxicity of aminopterin-monoclonal antibody conjugates: parameters for optimization of therapy. *Cancer Res.*, **50**, 6028–6033.

SANDS H, SHAH SA AND GALLAGHER BM. (1985). Vascular volume and permeability of human and murine tumors grown in athymic mice. *Cancer Lett.*, **27**, 15–21.

SANDS H, JONES PL, SHAH SA, PALME D, VESSELLA RL AND GALLAGHER BM. (1988). Correlation of vascular permeability and blood flow with monoclonal antibody uptake by human Clouser and renal cell xenografts. *Cancer Res.*, **48**, 188–193.

SATO N, GOTO T, HARANAKA K, SATOMI N, NARIUCHI H, MANO-HIRANO Y AND SAWASAKI Y. (1986). Actions of tumor necrosis factor on cultured vascular endothelial cells: morphological modulation, growth inhibition, and cytotoxicity. *J. Natl Cancer Inst.*, **76**, 1113–1121.

SAVAGE P, SO A, SPOONER RA AND EPENETOS AA. (1993). A recombinant single chain antibody interleukin-2 fusion protein. *Br. J. Cancer*, **67**, 304–310.

SHIRAI T, YAMAGUCHI H, ITO H, TODD CW AND WALLACE RB. (1985). Cloning and expression in *Escherichia coli* of the gene for human tumour necrosis factor. *Nature*, **313**, 803–806.

SLØRDAL L, MUENCH MO, WARREN DJ AND MOORE MAS. (1989). Radioprotection by murine and human tumor-necrosis factor: dose-dependent effects on hematopoiesis in the mouse. *Eur. J. Haematol.*, **43**, 428–434.

SMITH WW, ALDERMAN IM AND GILLESPIE RE. (1957). Increased survival in irradiated animals treated with bacterial endotoxins. *Am. J. Physiol.*, **191**, 124–130.

SMYTH MJ, PIETERSZ GA AND MCKENZIE IFC. (1987). Use of vasoactive agents to increase tumor perfusion and the antitumor efficacy of drug-monoclonal antibody conjugates. *J. Natl Cancer Inst.*, **79**, 1367–1373.

SONG CW AND LEVITT SH. (1970). Effect of X irradiation on vascularity of normal tissues and experimental tumor. *Radiology*, **94**, 445–447.

SPURR NK, DURBIN H, SHEER D, PARKAR M, BOBROW L AND BODMER WF. (1986). Characterization and chromosomal assignment of a human cell surface antigen defined by the monoclonal antibody AUA1. *Int. J. Cancer*, **38**, 631–636.

TARTAGLIA LA, WEBER RF, FIGARI IS, REYNOLDS C, PALLADINO Jr MA AND GOEDDEL DV. (1991). The two different receptors for tumor necrosis factor mediate distinct cellular responses. *Proc. Natl Acad. Sci. USA*, **88**, 9292–9296.

TAYLOR-PAPADIMITRIOU J, PETERSON JA, ARKLIE J, BURCHELL J, CERIANI RL AND BODMER WF. (1981). Monoclonal antibodies to epithelium-specific components of the human milk fat globule membrane: production and reaction with cells in culture. *Int. J. Cancer*, **28**, 17–21.

TRACEY KJ, BEUTLER B, LOWRY SF, MERRYWEATHER J, WOLPE S, MILSARK IW, HARIRI RJ, FAHEY III TJ, ZENTELLA A, ALBERT JD, SHIRES GT AND CERAMI A. (1986). Shock and tissue injury induced by recombinant human cachectin. *Science*, **234**, 470–474.

VAN DE WIEL PA, BLOKSMA N, KUPER CF, HOFHUIS FM AND WILLERS JM. (1989). Macroscopic and microscopic early effects of tumour necrosis factor on murine Meth A sarcoma, and relation to curative activity. *J. Pathol.*, **157**, 65–73.

VAN DE WIEL PA, BOUMA GJ, VAN DER PIJL A, WEITENBERG ES, LAM AW AND BLOKSMA N. (1990). Effect of tumour necrosis factor and lipid A on functional and structural vascular volume in solid murine tumours. *Br. J. Cancer*, **62**, 718–723.

WONG GHW AND GOEDDEL DV. (1988). Induction of manganous superoxide dismutase by tumor necrosis factor: possible protective mechanism. *Science*, **242**, 941–944.